# Referral of a Patient With Ocular Symptoms to the Stroke Clinic: Not Always the Usual Suspect!

**DOI:** 10.7759/cureus.53912

**Published:** 2024-02-09

**Authors:** Kayteck Ling, Saugata Das, Sanjay Vydianath

**Affiliations:** 1 Geriatric Medicine, University Hospitals of Derby and Burton NHS Foundation Trust, Burton-on-Trent, GBR; 2 Stroke Medicine, The Royal Wolverhampton NHS Trust, Wolverhampton, GBR; 3 Radiology, The Royal Wolverhampton NHS Trust, Wolverhampton, GBR

**Keywords:** rt-quic, perfusion mri, prion diseases, heidenhain variant, cjd

## Abstract

A 77-year-old male attended the stroke clinic as a delayed presentation of a stroke and was initially managed as an occipital stroke. He presented with a gradual decline in visual acuity with an initial suspicion of field deficit over a period of three to four months. He underwent extensive tests including imaging for a confirmatory diagnosis. He had a rapid deterioration of his vision, function, and cognition over a few weeks resulting eventually in death. The case highlights a rare variant of sporadic Creutzfeld-Jakob disease (sCJD), the Heidenhain Variant (HV-CJD). CJD is the commonest of human prion diseases. In HV-CJD, pathologic prions display demyelinating neurotropism for the occipital lobes resulting in visual changes and hallucinations.

## Introduction

Creutzfeld-Jakob disease (CJD) is a rare and fatal disease that causes progressive neurodegeneration and worsens rapidly over time. CJD is caused by an infectious protein called a prion, which accumulates at high levels in the brain and leads to spongiform encephalopathy causing irreversible damage to nerve cells [1]. Clinical presentation is highly variable, and often the first symptoms are cognitive or cerebellar. A conspicuous feature is rapid neuropsychiatric decline with death usually occurring within one year of symptom onset [1]. The Heidenhain variant of CJD (HV-CJD) is rare, comprising approximately 4.9% of all sporadic CJD (sCJD) that present with visuospatial symptoms and a more rapid decline compared to other forms [2].

## Case presentation

A 77-year-old male with a background of hypertension, type 2 diabetes mellitus, hypothyroidism, chronic obstructive pulmonary disease, and chronic right mastoiditis presented to his opticians with blurring vision and gradual bilateral decline in visual acuity over a period of three to four months. The opticians noted bilateral cataracts and suspected a possible left homonymous hemianopia and referred him to the ophthalmology clinic. The ophthalmologist made an onward referral to the stroke clinic in view of the suspected hemianopia.

He presented to the stroke clinic with no other objective deficit, apart from reduced visual acuity with reduced left-sided visual perception. He was clinically well and independent with his activities of daily living (ADL). 

**Figure 1:**
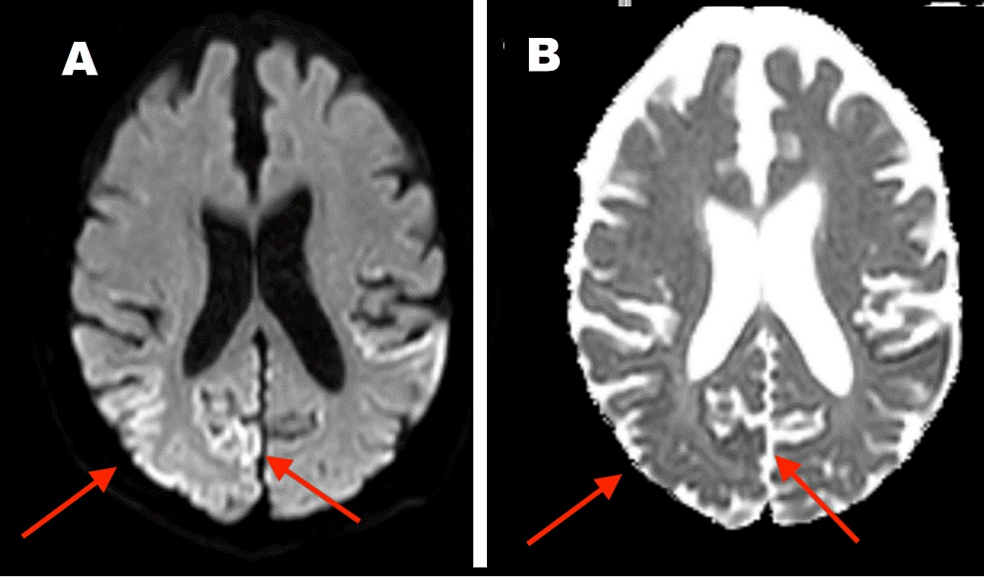
Initial MRI Brain shows cortical changes in the right occipital region (A) Diffusion-weighted imaging (DWI) sequence; (B) Apparent diffusion coefficient (ADC) sequence

An MRI brain suggested T2 hyperintensity in a cortical distribution predominantly in the right occipital lobe with diffusion restriction on the diffusion-weighted imaging (DWI) sequences and corresponding changes on the apparent diffusion coefficient (ADC) map (Figure 1). There was some suggestion of similar but less prominent changes in the left occipital lobe. 

He had an ultrasound Doppler of carotid arteries which did not show evidence of significant internal carotid artery disease and an ECG which was sinus rhythm at 60 beats per minute. The case was discussed in the neuroradiology meeting and the consensus was to manage it as an occipital stroke in view of his risk factors. He was started on clopidogrel and a statin while awaiting a repeat MRI of the brain in a months' time to reassess for any developing or established parenchymal changes. 

The patient’s family described a marked decline while waiting for the next appointment for his MRI brain. Within a month, from being independent with his ADLs, he required assistance for using the toilet, mobilising, and personal care. Two days prior to his MRI appointment, he developed abnormal posturing of his left hand and wrist with intermittent jerky movements perceived as ‘kicking in bed at night’ and developed visual hallucinations. Following the repeat MRI, the patient was admitted as an emergency admission directly to the stroke ward for further management.

**Figure 2:**
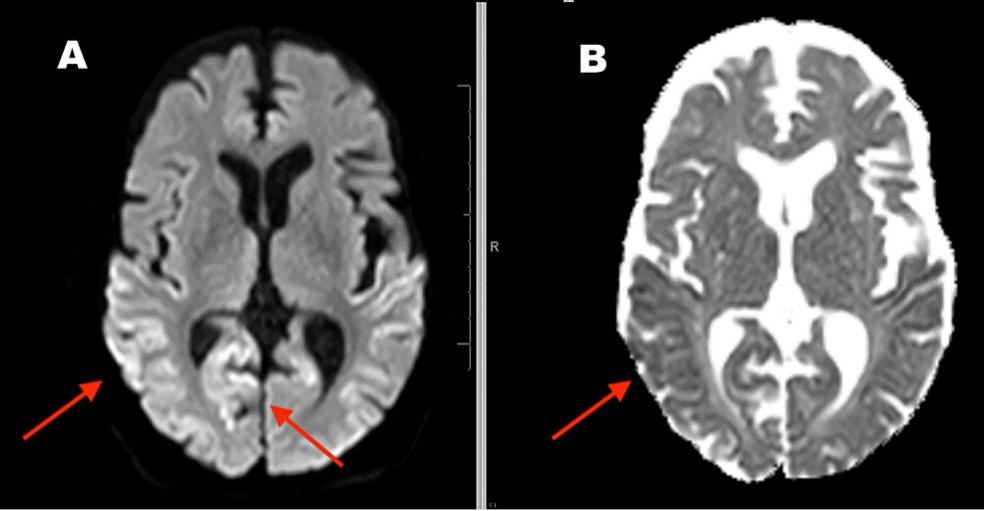
Follow-up MRI Brain after one month shows progression of right parietal and temporal regions with cortical changes in the DWI image (A) (A) Diffusion-weighted imaging (DWI) sequence; (B) Apparent diffusion coefficient (ADC) sequence

**Figure 3:**
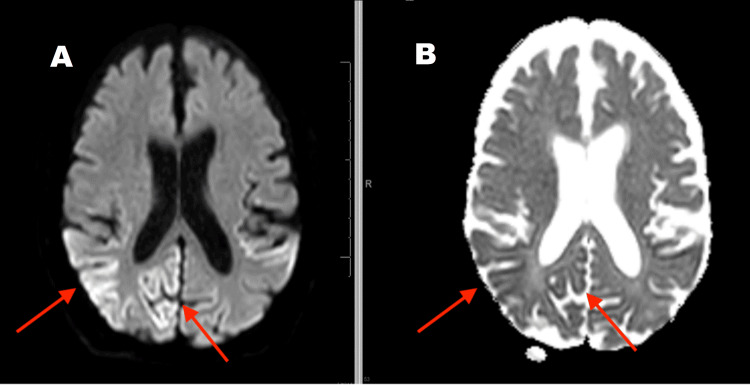
Follow-up MRI Brain after one month shows right occipital region "cortical ribboning" changes (A) Diffusion-weighted imaging (DWI) sequence; (B) Apparent diffusion coefficient (ADC) sequence

**Figure 4:**
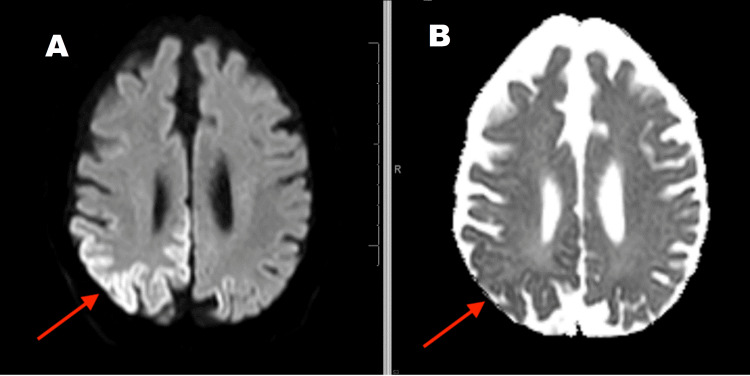
Follow-up MRI Brain after one month shows right superior parietal region "cortical ribboning" in the DWI image (A) and persistent ADC changes (B) (A) Diffusion-weighted imaging (DWI) sequence; (B) Apparent diffusion coefficient (ADC) sequence

The follow-up MRI (Figures 2-4) suggested extensive 'cortical ribboning' on the DWI imaging seen as cortical hyperintensity in the right temporal, parietal, and occipital regions, especially in the visual field areas. Focal cortical ribboning was seen in the paramedian cortex in both frontal lobes. Similar changes were seen in the left occipital and temporal lobes. Corresponding ADC changes were persistent. Clinical suspicion was sCJD at this point.

During his admission, EEG showed rhythmic, periodic sharp waves and triphasic waves. Cerebrospinal fluid (CSF) analysis suggested acellular fluid with protein of 0.61 with a positive real-time quaking-induced conversion (RT-QuIC). Other causes such as bacterial/viral infections, paraneoplastic causes, and autoimmune disease were ruled out. 

He was referred to the National CJD Research & Surveillance Unit in Edinburgh, Scotland. The specialised team conducted a local visit to review the patient. HV-CJD was diagnosed, which was associated with a rapid decline. Following a discussion about the natural progression of this devastating and incurable disease with the family, a palliative approach was agreed upon. He was discharged home with palliative care team support as this was deemed the preferred place for terminal phase care. He sadly passed away two weeks post discharge.

## Discussion

sCJD is the most well-known and accounts for approximately 90% of sporadic prion disease [1]. Although the exact aetiology of sCJD is unknown, it has been hypothesised that a mutation of a normal brain protein results in it becoming a prion. Iatrogenic CJD, variant CJD, and genetic CJD are some other subtypes of CJD [1]. Prions arise in the adult brain as a result of a mutation in the prion protein gene inherited from one's parents in genetic CJD, an extremely rare genetic illness [1]. Variant CJD is most likely caused by eating meat from a cow that has bovine spongiform encephalopathy, also known as "mad cow" disease [1]. Iatrogenic CJD is the term used to describe an infection that is inadvertently spread from a person with CJD through medical or surgical treatments.

In this case, there were no features suggestive of variant/iatrogenic CJD. The probability of genetic CJD was low due to age, illness progression, and absence of family history. The patient’s presentation fulfilled the criteria for HV-sCJD given the isolated visual disturbance at the onset of disease, cortical ribboning on MRI, CSF positive for RT-QuiC, and rapid clinical decline [3-5].

RT-QuIC is a CSF assay in which disease-associated prion protein (PrPSc) initiates a conformational transition in recombinant prion protein (recPrP), resulting in the formation of amyloid that can be monitored [6]. The sensitivity and specificity of RT-QuIC were 87-91% and 98-100%, respectively a noted series with a validation cohort [6,7]. Other CSF markers include tau protein levels (87% sensitive; 67% specific), and protein 14-3-3 levels (90% sensitive; 40% specific) [6,7].

In the current case, the follow-up MRI showed the progression of cortical lesions and persistent hyperintensity on DWI sequences after a month, which raised a strong suspicion. Typically, a lesion with hyperintensity on DWI and hypo-intensity on the ADC map is a characteristic MRI finding for an acute ischaemic infarction [8]. DWI increases during the first week of an acute stroke after symptom onset and decreases after two weeks [8,9]. Previous reports have documented that other than in an acute stroke setting, the significance of restricted diffusion is frequently not appreciated, resulting in missed or delayed diagnosis [8-10].

The majority of sCJD patients die within one year of symptom onset, and HV-CJD presents a more rapid decline with death, as demonstrated in our patient. Management remains supportive, and early referral to palliative care was key to management for the patient and his family [4,5].

## Conclusions

CJD is an extremely rare and deadly brain disease. It results in progressive brain damage that gets worse over time. Individuals diagnosed with CJD may present with rapid neurocognitive decline (dementia), athetosis, myoclonus, dysarthria, visual disturbances and/or muscle weakness and loss of mass (wasting). Our case highlights that although rare, HV-CJD is a relevant differential in patients with visual symptoms due to the involvement of the parieto-occipital cortex which is accompanied by myoclonus and rapid neurocognitive decline. CSF studies, EEG, and typical MRI features may help diagnosis while excluding other potentially treatable causes. Management remains supportive, and early referral to the National CJD Research and Surveillance Unit along with the involvement of the palliative care team was key to management for our patient and brought closure to his family.
